# Regulation of actin dynamics by WNT-5A: implications for human airway smooth muscle contraction

**DOI:** 10.1038/srep30676

**Published:** 2016-07-29

**Authors:** Tim Koopmans, Kuldeep Kumawat, Andrew J Halayko, Reinoud Gosens

**Affiliations:** 1 Department of Molecular Pharmacology, University of Groningen, The Netherlands; 2 Groningen Research Institute for Asthma and COPD (GRIAC), University of Groningen, The Netherlands; 3 Department of Physiology and Pathophysiology, University of Manitoba, Canada

## Abstract

A defining feature of asthma is airway hyperresponsiveness (AHR), which underlies the exaggerated bronchoconstriction response of asthmatics. The role of the airway smooth muscle (ASM) in AHR has garnered increasing interest over the years, but how asthmatic ASM differs from healthy ASM is still an active topic of debate. WNT-5A is increasingly expressed in asthmatic ASM and has been linked with Th2-high asthma. Due to its link with calcium and cytoskeletal remodelling, we propose that WNT-5A may modulate ASM contractility. We demonstrated that WNT-5A can increase maximum isometric tension in bovine tracheal smooth muscle strips. In addition, we show that WNT-5A is preferentially expressed in contractile human airway myocytes compared to proliferative cells, suggesting an active role in maintaining contractility. Furthermore, WNT-5A treatment drives actin polymerisation, but has no effect on intracellular calcium flux. Next, we demonstrated that WNT-5A directly regulates TGF-β1-induced expression of α-SMA via ROCK-mediated actin polymerization. These findings suggest that WNT-5A modulates fundamental mechanisms that affect ASM contraction and thus may be of relevance for AHR in asthma.

Asthma is a disease characterized by chronic inflammation of the large and small airways, and estimated to affect 235 million people worldwide^1^. A defining feature of asthma is airway hyperresponsiveness (AHR), which underlies the exaggerated bronchoconstriction response of asthmatics. While it is clear that the airway smooth muscle (ASM) is essential in mediating airway constriction typical of AHR in asthma, there remains much debate concerning the root cause of altered ASM contraction and AHR. The mass of ASM is generally greater in the airway wall of asthmatics due to cellular hyperplasia or hypertrophy, but whether or not the ASM behaves differently in terms of contractility is still controversial. Asthmatic ASM does not appear to produce more force per cell^2^,^3^,^4^, but evidence from isolated human ASM cells suggests that asthmatic ASM exhibits greater shortening capacity^5^,^6^,^7^. Not all studies have reproduced this finding in whole ASM tissue^2^,^4^,^8^, suggesting that the mechanical properties of the surrounding extracellular tissue matrix may also be altered in asthma. In this regard it is notable that the ASM of asthmatics does appear to respond differently to strain induced by tidal breathing. In healthy individuals, deep inspiration causes a bronchodilatory effect that can prevent or alleviate airway narrowing, whereas this response is largely absent in asthmatics^9^,^10^,^11^,^12^,^13^.

Insights into the mechanisms that may underpin altered contractility of ASM are diverse and not fully conclusive. Some studies^5^,^14^,^15^, but not all^6^,^16^, show increased expression of myosin light chain kinase (MLCK) in ASM samples from asthmatics. Similarly, there may be increased abundance of myosin heavy chain in asthmatic ASM^15^, however not all studies prove this to be the case^5^,^16^. Other changes in asthmatic ASM cells may reside in the filamentous organization of the contractile apparatus as it has been proposed that the myosin thick filaments are less prone to disruption following mechanical strain^4^,^17^. Additionally, there is evidence that actin filament polymerization may underlie the altered behaviour of asthmatic ASM^18^,^19^. In this case, whereas mechanical stretch leads to shortening actin filaments and derangement of myosin filament assembly, in asthmatic ASM longer actin filaments are associated with refractoriness of thick myosin filament derangement following mechanical stretch, thus supporting greater force-generating capacity.

The WNT (wingless-integrase-1) signalling pathway consists of a family of secreted glycoproteins that are heavily modified before entering the extracellular space^20^,^21^. They regulate a plethora of functions, from embryonic development to the maintenance of adult tissue homeostasis^22^. WNT is broadly categorized into β-catenin-dependent (canonical) and -independent signalling (non-canonical). Non-canonical WNT signalling is actively utilized by airway smooth muscle. In ASM cell lines, transforming growth factor beta (TGF-β1) requires WNT-5A for the production of extracellular matrix (ECM) components such as fibronectin and collagen. Here, WNT-5A acts upon classical non-canonical mediators, increasing c-Jun N-terminal kinase (JNK) phosphorylation to enhance TGF-β1-mediated production of fibronectin^23^.

We have shown that WNT-5A protein expression is increased in ASM cells isolated from asthmatics compared to non-asthmatic individuals^23^ and WNT-5A expression has been associated with Th2-high asthma^24^. Historically, WNT-5A is known for its role in calcium homeostasis. Ectopic expression of WNT-5A increases the frequency of cytosolic calcium waves in Xenopus embryos^25^,^26^, due to activation of Ca^2+^/calmodulin-dependent protein kinase II (CamKII) and protein kinase C (PKC)^27^,^28^. In addition, WNT-5A directly promotes cellular adhesion and directional migration through effects on dynamics of cytoskeletal filaments and microtubules^29^. Here we test the hypothesis that WNT-5A modulates the contractile response of ASM and actin dynamics. We found that WNT-5A is able to increase maximum isometric contraction in isolated bovine tracheal smooth muscle strips. We provide evidence that this is mediated by rearranging actin filaments, as we found that in cultured human ASM cells, WNT-5A increases the F/G-actin actin, while no changes in expression of alpha-smooth muscle actin (α-SMA) or [Ca^2+^] handling were evident. Inhibition of Rho kinase (ROCK-I) completely abolished effects of WNT-5A in both human ASM as well as bovine smooth muscle strips. Finally, we found that WNT-5A knock down attenuates TGF-β1-induced α-SMA expression and that exogenous WNT-5A synergistically increased TGF-β1-induced α-SMA expression in human ASM cells.

## Results

### WNT-5A increases isometric contraction in bovine tracheal smooth muscle

**Figure 1:**
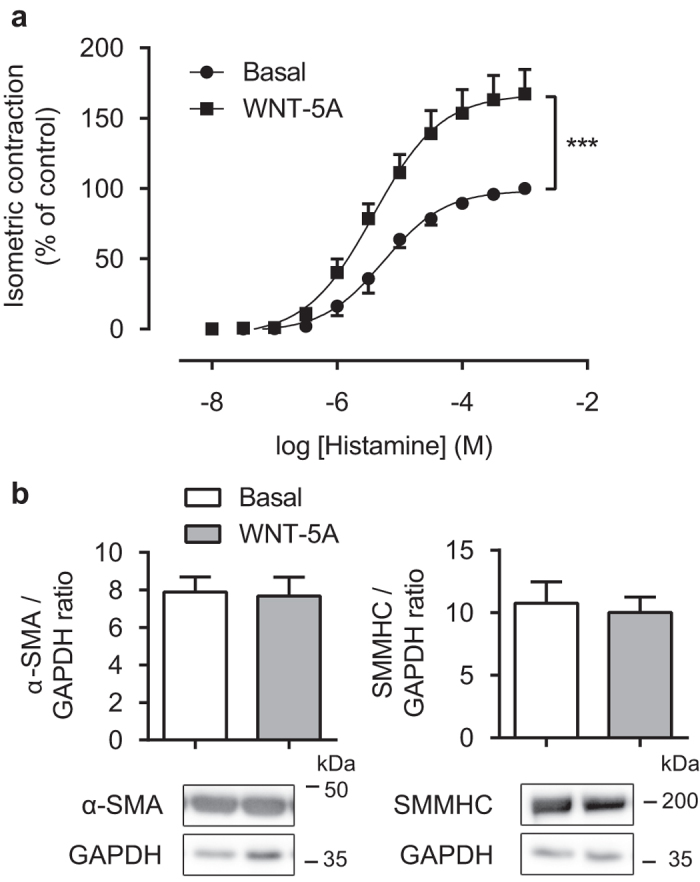
WNT-5A potentiates histamine-induced maximum isometric tension. (**a**) Organ-cultured bovine tracheal smooth muscle strips were pre-incubated with WNT-5A (500 ng/mL) for 48 hours and a cumulative dose-response curve to histamine for the maximum isometric tension was constructed. Data represents five independent experiments, each performed in duplicate. (**b**) Alpha smooth muscle actin and smooth muscle myosin heavy chain immunoblot of lysates from bovine tracheal smooth muscle strips, normalised against GAPDH. Strips were pre-incubated with WNT-5A (500 ng/mL) for 48 hours. Data represents six independent experiments. Cropped images are shown. Full-length blots are presented in Supplementary Fig. 1. α-SMA and GAPDH blots were derived from the same gel; SMMHC was run on a separate gel. Data is expressed as the mean ± SEM.

Non-canonical WNT signalling is implicated in both cytoskeletal reorganization as well changes in calcium homeostasis, both of which may facilitate smooth muscle contraction. We hypothesized that WNT-5A increases ASM contraction by acting upon one of these systems. To test this, we pre-incubated recombinant WNT-5A for 48 hours with isolated bovine tracheal smooth muscle strips and subjected them to isometric tension measurements using histamine as the contractile agonist. We found that the maximal histamine-induced contractile force (E_max_) was significantly higher for the muscle strips cultured with WNT-5A (E_max_ = 167.4 ± 16.7% of control; Fig. 1a). No difference in the sensitivity to histamine was found (pEC_50_ = 5.3 ± 0.14 and 5.44 ± 0.12, p=0.53, for the control and WNT-5A-treated group respectively). Interestingly, these changes were not associated with increases in smooth muscle myosin heavy chain (sm-MHC) or α-SMA protein abundance (Fig. 1b).

### WNT-5A does not affect calcium handling in human airway smooth muscle cells

**Figure 2:**
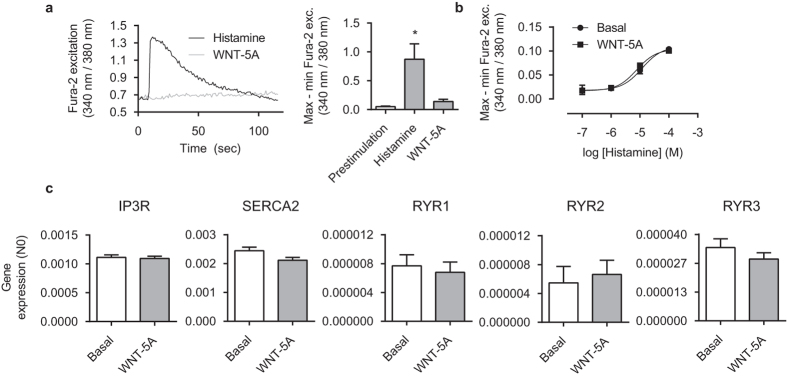
Calcium handling is not affected by WNT-5A. (**a**) Representative Fura-2 traces (left) and quantification (right) of intracellular calcium (Ca^2+^_i_) changes with respect to time of immortalized human airway smooth muscle cells exposed to WNT-5A (500 ng/mL) or histamine (10^−4^ M). 30–40 cells were simultaneously measured in the presence of extracellular Ca^2+^ and collectively determined the response. Data represents four independent experiments. *vs pre-stim. (**b**) Airway smooth muscle cells were pre-incubated with WNT-5A (500 ng/mL) for 48 hours and a dose-response curve to histamine for the maximum Ca^2+^_i_ peak response was constructed. Data represents four independent experiments. (**c**) mRNA of airway smooth muscle cells pre-incubated with WNT-5A (500 ng/mL) for 24 hours was isolated and subjected to RT-qPCR. Data represents five independent experiments. Data is expressed as the mean ± SEM.

To elucidate the mechanisms contributing to the WNT-5A-enhanced contractile response we next used cultured human bronchial smooth muscle cell lines to assess the impact of WNT-5A on intracellular calcium release using fluorescence microscopy with the Fura-2 calcium indicator. We found that WNT-5A alone was insufficient to induce release of intracellular calcium. Conversely, as expected histamine exposure elicited rapid increase in cytosolic calcium (16.8 ± 0.27 fold of pre-stim; Fig. 2a). Pre-incubation of cells with WNT-5A (48 hours) had no impact on histamine-induced calcium release (Fig. 2b). Consistent with these observations WNT-5A pre-incubation had no effect on the gene expression profile for endoplasmic reticulum calcium channels and receptors: IP3R, SERCA2, nor any ryanodine receptor isoforms were differentially expressed following WNT-5A treatment (Fig. 2c). Together, the data suggests that, contrary to some non-smooth muscle cell types, WNT-5A has no (in)direct effect on calcium handling in cultured human ASM cells.

### WNT-5A drives actin cytoskeletal reorganization

**Figure 3:**
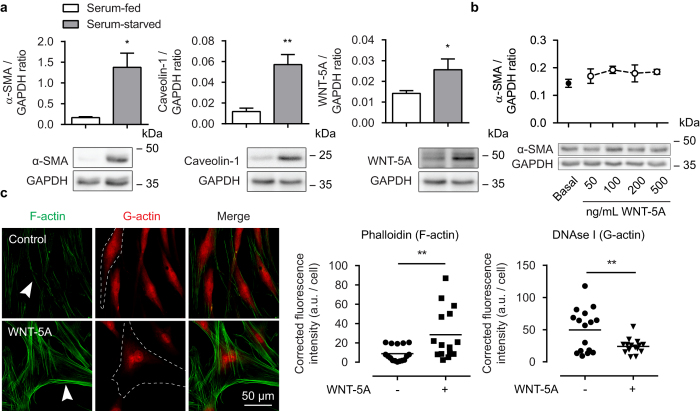
Involvement of WNT-5A in the regulation of alpha smooth muscle actin. (**a**) Alpha smooth muscle actin, caveolin-1 and WNT-5A immunoblot of lysates from cultured human airway smooth muscle cells, normalised against GAPDH. Cell were grown to 50% confluence in serum-enriched (10% FBS) DMEM (serum-fed group) or grown to confluence and then serum-deprived in Ham’s F12 supplemented with ITS (serum-starved group) for 7 days. Cropped images are shown. Full-length blots are presented in Supplementary Fig. 1. α-SMA/GAPDH and caveolin-1/WNT-5A/GAPDH were derived from the same gel. Data represents four independent experiments. *vs serum-fed. (**b**) Airway smooth muscle actin immunoblot as performed in (**a**). Cells were treated with WNT-5A for 24 hours. Full-length blots are presented in Supplementary Fig. 1. Data represents three independent experiments. (**c**) Representative immunofluorescent images of a Phalloidin (F-actin, green) and DNAse I (G-actin, red) staining of airway smooth muscle cells exposed to WNT-5A (200 ng/mL) for 2 hours, and the corresponding quantification. White arrowhead points to filamentous actin. Dashed line represents a single cell boundary. Horizontal line represents the mean. Data is expressed as the mean ± SEM.

We next investigated links between WNT-5A and changes in the actin cytoskeleton. We first compared abundance of endogenous WNT-5A in contractile phenotype and synthetic/proliferative phenotype human ASM, using 7-day serum-starved ASM cells (contractile phenotype) and serum-fed cultures (synthetic/proliferative phenotype) as we have described^30^. As expected, serum-starvation induced accumulation of α-SMA and caveolin-1 (8.3 ± 1.5 fold of serum-fed and 6.0 ± 1.4 fold of serum-fed respectively; Fig. 3a). Interestingly, WNT-5A expression was also increased with prolonged serum starvation (1.8 ± 0.28 fold of serum-fed; Fig. 3a). We next investigated whether increasing concentrations of WNT-5A might affect α-SMA expression in human ASM cells, but we saw no evidence for a change in α-SMA abundance with increasing recombinant WNT-5A concentration (24 hours) (Fig. 3b).

We next investigated whether WNT-5A induced changes in the organizational structure of the actin cytoskeleton. We measured the effects of WNT-5A exposure in cultured human ASM cells by co-labelling F- and G-actin with fluorescently-conjugated phalloidin and DNAse I, respectively. Microscopy analysis revealed that treatment with recombinant WNT-5A increased polymerized actin, as revealed by increased phalloidin staining and reduced DNAse I labelling (Fig. 3c). Together, these data suggest WNT-5A drives actin cytoskeletal reorganization in human ASM and is associated with contractile phenotypic changes.

### ROCK-I activation underlies WNT-5A-driven F-actin formation

**Figure 4:**
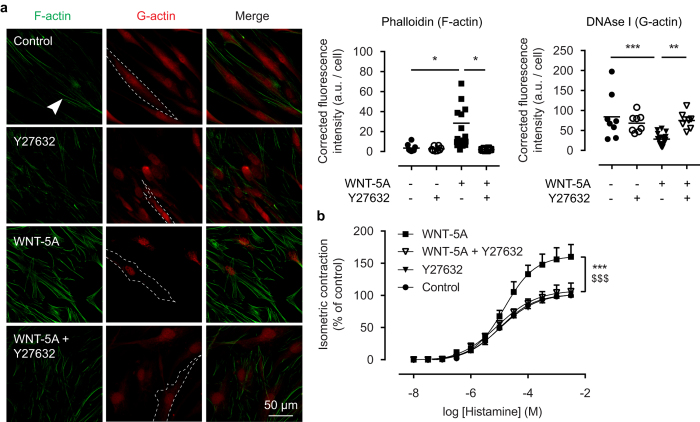
ROCK activation underlies WNT-5A-induced actin polymerisation. (**a**) Representative immunofluorescent images of a Phalloidin (F-actin, green) and DNAse I (G-actin, red) staining of airway smooth muscle cells exposed to WNT-5A (200 ng/mL) for 2 hours in the presence or absence of Y27632 (1.0 μM), and the corresponding quantification. White arrowhead points to filamentous actin. Dashed line represents a single cell boundary. Horizontal line represents the mean. (**b**) Organ-cultured bovine tracheal smooth muscle strips were pre-incubated with WNT-5A (500 ng/mL) and/or Y27632 (1.0 μM) for 48 hours and a cumulative dose-response curve to histamine for the maximum isometric tension was constructed. *vs Control, $ vs WNT-5A + Y27632. Data represents five independent experiments, each performed in duplicate. Data is expressed as the mean ± SEM.

RhoA-dependent activation of ROCK-I induces actin polymerization and stress fiber formation in many cell types^31^,^32^, among which airway smooth muscle. Thus, we treated human ASM cells with the highly selective ROCK inhibitor, Y27632, and thereafter performed a phalloidin and DNAse I staining. While Y27632 alone had little effect on baseline labelling of F-actin and G-actin, it entirely prevented accumulation of phalloidin-labelled F-actin that was otherwise induced by WNT-5A (Fig. 4a). To integrate these findings with our bovine model for smooth muscle contraction, we treated isolated bovine tracheal smooth muscle strips with WNT-5A and Y27632 for 48 hours, and then subjected them to isometric tension measurements. Again, we found that while Y27632 alone had no effect on E_max_ or sensitivity to histamine, it completely abolished the effects of WNT-5A on histamine-induced force generation (Fig. 4b).

### WNT-5A is required for TGF-β1-induced actin polymerization

**Figure 5:**
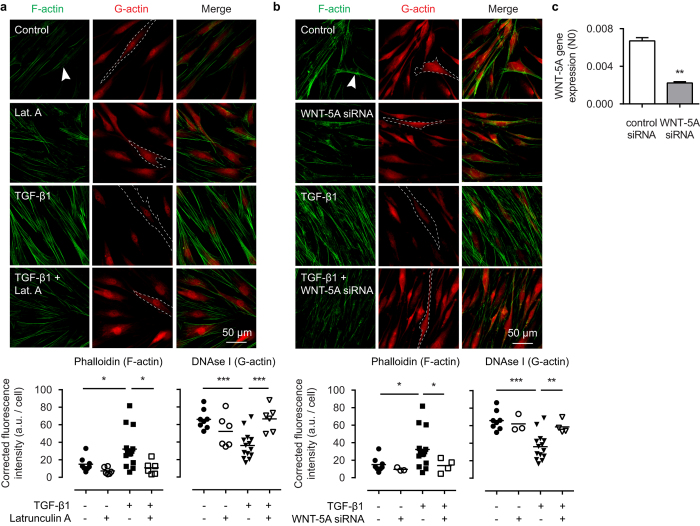
WNT-5A mediates TGF-β1 induced actin polymerization. Representative immunofluorescent images of a Phalloidin (F-actin, green) and DNAse I (G-actin, red) staining of airway smooth muscle cells exposed to TGF-β1 (2 ng/mL) for 48 hours in the presence or absence of (**a**) latrunculin A (0.1 μM), or (**b**) WNT-5A-specific siRNA. White arrowhead points to filamentous actin. Dashed line represents a single cell boundary. Horizontal line represents the mean. (**c**) mRNA of airway smooth muscle cells pre-incubated with WNT-5A siRNA or control siRNA (30 pmol) for 36 hours was isolated and subjected to RT-qPCR. *vs control siRNA. Data represents three independent experiments. Data is expressed as the mean ± SEM.

We have shown that WNT-5A is under direct control of TGF-β1 in the regulation of extracellular matrix proteins in human ASM cells^23^,^33^. Because TGF-β1 is also known to have an impact on cytoskeletal organization, we investigated whether TGF-β1 requires WNT-5A for its effects on filamentous actin dynamics. As expected, in human ASM TGF-β1 strongly induced F-actin accumulation with a concomitant reduction of cytosolic G-actin, as shown by phalloidin and DNAse I staining. This effect could be completely eliminated by addition of latrunculin A, a potent inhibitor of actin polymerization that stabilizes G-actin (Fig. 5a). To address the role of WNT-5A in this pathway, we inhibited WNT-5A expression by means of WNT-5A-specific small-interfering RNA (siRNA), which reached 67 ± 6% knockdown efficiency (Fig. 5c). Interestingly, silencing WNT-5A expression using siRNA effectively blocked F-actin formation induced by TGF-β1 (Fig. 5b), underlying the regulatory role of WNT-5A in TGF-β1-induced actin polymerization.

### Actin polymerization induced by WNT-5A is essential for TGF-β1-mediated actin expression

**Figure 6:**
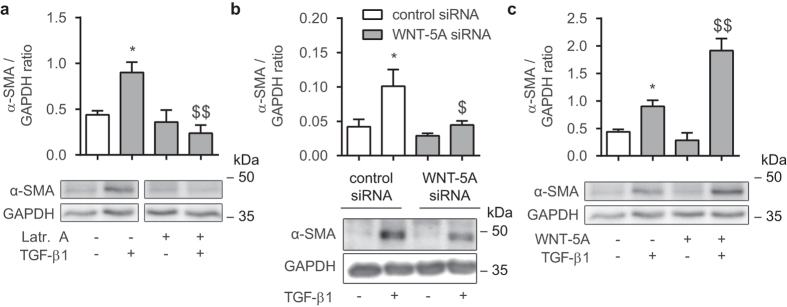
TGF-β1-induced actin expression utilises WNT-5A. (**a**) Alpha smooth muscle actin immunoblot of lysates from cultured human airway smooth muscle cells, normalised against GAPDH. Cells were treated with TGF-β1 (2 ng/mL) for 48 hours in the presence or absence of latrunculin A (0.1 μM). Cropped images are shown. Full-length blots are presented in Supplementary Fig. 1. α-SMA and GAPDH were derived from the same gel. Data represents four independent experiments. *vs control, $ vs TGF-β1. (**b**) Alpha smooth muscle actin immunoblot performed as in (**a**). Cells were transfected with a WNT-5A-specific siRNA construct or scrambled negative control siRNA and treated with TGF-β1 (2 ng/mL) for 48 hours. Full-length blots are presented in Supplementary Fig. 1. Data represents six independent experiments. *vs control siRNA, $ vs control siRNA + TGF-β1. (**c**) Alpha smooth muscle actin immunoblot performed as in (**a**). Cells were treated with TGF-β1 (2 ng/mL) for 48 hours in the presence or absence of WNT-5A (200 ng/mL). Full-length blots are presented in Supplementary Fig. 1. Data represents three independent experiments. *vs control, $ vs TGF-β1. Data is expressed as the mean ± SEM.

Many smooth muscle-specific promoter regions are under direct control of actin-binding proteins, and as such, increasing actin polymerization can enhance the promoter activity of these genes, for example α-SMA^34^. We investigated whether F-actin formation induced by WNT-5A is required for the transcriptional control of α-SMA (a known target gene for TGF-β1). To confirm that actin cytoskeletal reorganization is required for α-SMA expression, we treated human ASM cells with TGF-β1 and latrunculin A. Indeed, we found that latrunculin A completely blocked induction of α-SMA protein accumulation following exposure to TGF-β1 (48 hours) (0.26 fold of TGF-β1; Fig. 6a). To extend on these findings we treated our cell cultures with WNT-5A siRNA and found that this was associated with marked attenuation of α-SMA protein accumulation induced by TGF-β1 (0.44 fold of TGF-β1; Fig. 6b). In line with this, addition of recombinant WNT-5A with TGF-β1 resulted in a seemingly synergistic effect, where α-SMA abundance reached higher levels than the sum of the treatments alone (2.12 fold of TGF-β1; Fig. 6c). These data suggest that WNT-5A induced polymerization is critical for TGF-β1-mediated induction of α-SMA.

## Discussion

In this study we explored the role of non-canonical WNT signalling, focusing on the well-studied family member WNT-5A, in the regulation of cytoskeletal reorganization, mobilization of intracellular calcium and airway smooth muscle contraction. We found that in bovine tracheal smooth muscle strips, pre-treatment with WNT-5A increases maximum isometric tension induced by histamine, while there was no change in the abundance of α-smooth muscle actin or smooth muscle myosin heavy chain. It appears that modulation of actin dynamics may underlie these results, as WNT-5A was sufficient to increase the presence of filamentous actin via activation of ROCK-I, but was without effect on calcium release or endoplasmic reticulum proteins that regulate calcium handling, in cultured human airway smooth muscle cell lines. In addition, blocking ROCK-I with Y27632 also diminished WNT-5A effects on histamine-induced bovine smooth muscle contraction. We further observed that WNT-5A is preferentially expressed in human ASM cells of a contractile phenotype, suggesting an association with contractility. To our knowledge, this is the first study that has implicated WNT signalling in airway smooth muscle contraction. There are no reports prior to ours that reveal a role for WNT-5A in airway smooth muscle actin cytoskeletal reorganization, however, a number of publications describing use of other model systems corroborate our findings. In a study on the development and elongation of the small intestine of mice, WNT-5A^−/−^ mice displayed reduced smooth muscle thickness compared to wild type littermates. Also the number of subepithelial α-SMA-positive fibroblasts was lowered in WNT-5A mutant embryos^35^. In another study on the female reproductive tract of mouse pups, though smooth muscle cell layers developed normally in WNT-5A^−/−^ mutants they were thinner than in wildtype mice^36^. In cardiac development on the other hand, WNT-5A^−/−^ and WNT-11^−/−^ double knock-out mice showed no differences in the mRNA levels for α-SMA or the actin-associated smooth muscle marker SM-22^37^, suggesting that WNT-5A effects on cytoskeletal protein expression may be tissue-specific.

In our study, neither direct exposure to WNT-5A nor pre-incubation invoked any change in calcium handling. These findings are interesting, as historically the Frizzled-2 receptor has been ascribed a role in regulating calcium mobilization^38^. At the same time, the Frizzled-2 receptor is one of the most highly expressed Frizzled isoforms in both human airway smooth muscle^23^ as well as in MRC-5 human lung fibroblasts^39^. We have also shown that WNT-5A can activate NFAT downstream signalling in human ASM, implying a role for calcium^40^. The nature of the actual downstream event that regulates calcium signalling may be dependent on the presence of additional receptor complexes, such as the receptor tyrosine kinase-like orphan receptor 2 (ROR2) that is critically involved in non-canonical WNT signalling. ROR2 is present in very low numbers under basal conditions in airway smooth muscle cells^23^.

We show that Rho kinase activation is required for WNT-5A-induced actin cytoskeletal reorganization and effects on bovine smooth muscle contraction. In this study we did not fully explore the potential underlying mechanisms for this effect, however, there is an extensive body of work on the regulation of smooth muscle differentiation, characterized by the increased expression of smooth muscle specific marker genes, including α-SMA. Many smooth muscle genes contain CArG *cis*-elements (CC(A/T)_6_GG) that are under direct control of the transcription, factor serum response factor (SRF)^41^,^42^,^43^. SRF activation is dependent on actin dynamics and depletion of the G-actin pool is permissive for its activation^44^, in part via Rho kinase-dependent effects^34^. SRF-dependent transcription is also controlled by its interaction with other transcription factors and co-activators, including myocardin-related transcription factors (MRTFs) that interact with G-actin. Indeed Rho signalling promotes actin polymerization, thus reducing G-actin pools and permitting nuclear localization of MRTF by limiting G-actin association to the amino-terminal RPEL domain of MRTF^45^. Of relevance to our work, TGF-β1 can signal through the Rho-actin-MRTF axis^46^,^47^, and we show that pharmacologic inhibition of Rho kinase prevented WNT-5A-induced F-actin formation and bovine tracheal smooth muscle contraction. Thus, our findings suggest that WNT-5A signaling may engage the MRTF-SRF relay to regulate expression of α-SMA.

In this study we report that TGF-β1-induced expression of α-SMA in human ASM cells is dependent on the activation of WNT-5A signaling, relying on mechanisms that modulate the actin cytoskeleton. However, WNT-5A alone is not sufficient to increase abundance of α-SMA. These findings are consistent with the notion that TGF-β1 recruits additional factors beyond WNT-5A to regulate α-SMA expression. Apart from TGF-β1-responsive CArG elements in the α-SMA promoter, there is also a TGF-β1 control element (TCE) that confers TGF-β1 responsiveness in both smooth muscle cells and fibroblasts^41^,^48^. Additionally, a Smad-binding element (SBE) is present in the α-SMA promoter, and in fibroblasts, α-SMA expression is elevated following transfection with a Smad-3-expressing plasmid^49^. In epithelial cells Smad signaling is associated with epithelial-to-mesenchymal transition (EMT) that includes expression of α-SMA. Inhibition of Smad-3 by a virally induced Smad-3 double negative blocks TGF-β1-induced EMT, demonstrating an essential role for this pathway in TGF-β1-induced α-SMA expression^50^,^51^. Lack of activation of these promoter sites following exposure to WNT-5A alone may underlie its inability to induce α-SMA expression in human ASM.

It has been postulated that increased expression of α-SMA may contribute to the lack of effects caused by deep inspiration in individuals with asthma. However, to date unequivocal evidence for differences in total expression of αSMA in asthmatics and non-asthmatics is lacking^15^,^16^. A lot of effort has been directed towards finding candidate markers of the contractile machinery that are possibly changed in asthma, but perhaps an alternative attempt would be to direct our attention towards proteins that are not thought to be directly involved in contractile function. WNT-5A expression is increased in ASM cells isolated from mild to moderate asthmatics^23^ and this is associated with Th2-high asthma^24^. Even in the absence of changes directly related to contractile function, based on our findings changes in the expression of WNT-5A may affect ASM contractility and contribute to airway hyperresponsiveness.

In conclusion, we show that non-canonical WNT signalling via WNT-5A is a potent driver of actin cytoskeletal reorganization, shifting the F/G actin ratio in favour of filamentous actin. These findings result in increased maximum force generation following WNT-5A incubation in bovine tracheal smooth muscle strips. In addition, WNT-5A is actively utilised by TGF-β1, regulating TGF-β1-induced expression of α-SMA via ROCK-mediated actin polymerization. These findings may be of relevance for AHR in asthma.

## Materials and Methods

### Cell culture

Three human bronchial smooth muscle cell lines, immortalized by stable expression of human telomerase reverse transcriptase (hTERT), were used for all experiments. The primary cultured human bronchial smooth muscle cells used to generate each cell line were prepared as our laboratory has previously described from macroscopically healthy segments of second- to fourth-generation main bronchus obtained after lung resection surgery from patients with a diagnosis of adenocarcinoma^52^. Patients gave informed consent for secondary use of resected material for research purposes. All procedures and consent forms were approved by the Human Research Ethics Board (University of Manitoba) and in accordance with local and national guidelines. Up to 30^th^ passage cells were used for all experiments. Cells were grown in uncoated 100/20 mm tissue culture dishes (GBO, #664160) in Dulbecco’s Modified Eagle’s Medium (DMEM) (GIBCO, #42430-082) supplemented with 200 units/mL Penicillin-Streptomycin (GIBCO, #15070-063), 2.5 μg/mL antimycotic (GIBCO, #15290-026) and 10% vol/vol Fetal Bovine Serum (FBS) (Thermo Scientific, #SV30180.03).

### Antibodies and chemicals

The following antibodies were used: GAPDH (western blot 1:3000, Santa Cruz, #sc-47724), α-smooth muscle actin (western blot 1:1000, immunohistochemistry 1:100, Abcam, #ab5694), smooth muscle myosin heavy chain (western blot 1:400, Thermo Scientific, #MS1438), Caveolin-1 (western blot 1:1000, Santa Cruz, #sc-894), WNT-5A (western blot 1:500, Abcam, #ab72583), peroxidase-conjugated anti-mouse IgG (western blot 1:3000, Sigma-Aldrich, #A9044), peroxidase-conjugated anti-rabbit IgG (western blot 1:3000, Sigma-Aldrich, #A0545).

Other reagents used include the following: recombinant TGF-β1 (R&D Systems, #240-B), recombinant WNT-5A (R&D Systems, #645-WN), WNT-5A siRNA (Qiagen, #SI00051779), Negative control siRNA (Qiagen, #SI03650318), X-tremeGENE siRNA transfection reagent (Roche, #04476093001), Alexa Fluor^®^ 488-conjugated Phalloidin (Molecular Probes, #A12379), Alexa Fluor^®^ 594-conjugated Deoxyribonuclease I (Molecular Probes, #D12372), ProLong^®^ Gold Antifade Mountant (Molecular Probes, #P36930), Fura-2 AM (Molecular Probes, #F-14185), Histamine (Sigma Aldrich, #H7250), Bovine Serum Albumin (Sigma Aldrich, #A7030), Fetal Bovine Serum (FBS) (Thermo Scientific, #SV30180.03), Y-27632 (Tocris, #1254) and latrunculin A (Tocris, #3973). All other chemicals were of analytical grade.

### Western blot analysis

Cells were washed with PBS and incubated with RIPA lysis buffer (65 mM Tris, 155 mM NaCl, 1% Igepal CA-630, 0.25% sodium deoxycholate, 1 mM EDTA, pH 7.4, and a mixture of protease inhibitors: 1 mM Na_3_VO_4_, 1 mM NaF, 10 μg/ml leupetin, 10 μg/ml Pepstatin A, 10 μg/ml Aprotinin). Cells were then scraped from the plate and kept on ice for 15 min. Lysates were vortexed vigorously and finally centrifuged for 10 min at 10.000 g. Protein content of the supernatant fractions was determined with a BCA protein assay kit (Thermo Scientific, #23225) and subsequently subjected to SDS-PAGE, using 10% running gels. Separated proteins were transferred to PVDF membranes (Carl Roth, 0.45 μm, #T830.1), which were then blocked with ROTI^®^-Block blocking solution (Carl Roth, #A151.2) for 2 hours at room temperature. Membranes were incubated with primary antibodies overnight at 4 °C in TBST (50 mM Tris-HCl, 150 mM NaCl, 0.05% (w/v) Tween-20, pH 7.4). The next day, after washing in TBST, membranes were incubated with HRP-conjugated secondary antibody for 2 hours at room temperature. Finally, blots were developed using enhanced chemiluminescence substrate (Perkin Elmer, #NEL105001EA). Digital images were quantified by densitometry using LI-COR Image Studio Lite software.

### RT-qPCR

Cells were washed with PBS and incubated with lysis buffer before being scraped from the plate. Isolation of mRNA was performed with a NucleoSpin^®^ RNA isolation kit (Macherey-Nagel, # 740955.250) according to the manufacturer’s instructions. Equal amounts of cDNA were synthesized using AMV reverse transcriptase (Promega, #A3500) and diluted 15 times with RNAse-free ddH_2_O. Quantitative real-time PCR was performed on an Illumina Eco Real-Time PCR system using SYBR green as the DNA binding dye (Roche, #04913914001). PCR cycling was performed with denaturation at 94 °C for 30 sec, annealing at 60 °C for 30 sec and extension at 72 °C for 30 sec for 45 cycles. Analysis of RT-qPCR data was done using LinRegPCR analysis software^53^,^54^. 18S ribosomal RNA, B2M and RPL13A were used as reference loci for accurate normalization of the RT-qPCR data. Primer sequences are listed in Table 1. Total RNA yield was determined with a NanoDrop ND-1000 spectrophotometer and samples were normalized accordingly.

### Immunofluorescence

Cells were cultured in Lab-Tek 8-chambered coverglass slides (Thermo Scientific, #155409). After treatment, cells were washed in warm PBS and fixed in 4% paraformaldehyde (PFA) plus 4% sucrose in PBS for 15 min. Cells were then incubated with PBS containing 0.3% Triton X-100 for 2 min and then blocked in 5% BSA in PBS for 1 hour at RT. After blocking, cells were incubated with Alexa Fluor 488-conjugated Phalloidin (1:500) and Alexa Fluor^®^ 594-conjugated Deoxyribonuclease I (1:500) diluted in 1% BSA in PBS for 1 hour at RT. Finally, cells were washed in ddH_2_O and mounted with ProLong^®^ Gold antifade. Sections were imaged using the TissueFAXS imaging system (TissueGnostics).

Mean fluorescence intensity of phalloidin and DNAse I stainings were digitally analysed with ImageJ. Following a 300 px background subtraction, images were thresholded and the integrated density per area from the appropriate channel was calculated, using the perimeter of the cell boundary. For each data set, each replicate represents the mean of eight analysed cells. Values are expressed as arbitrary units (a.u.).

### [Ca^2+^]_i_ measurements

Cells were loaded with Fura-2 in the presence of Pluronic acid (GIBCO, #24040-032) by incubation at 37 °C for 1 h in 5 μM Fura-2 AM in Ca^2+^ (1.3 mM)-containing Hanks’ buffered salt solution without phenol red (Molecular Probes, #14065) and additionally buffered with 20 mM HEPES (Sigma Aldrich, #H3375) (HHBSS, pH 7.4). The cells were then washed and allowed to incubate at room temperature for 30 min to allowed for complete de-esterification of the Fura-2 AM. Cells were visualized with an inverted microscope equipped with fluorescence optics and a 40x objective lens. Fluorescence was detected with a computer controlled monochromatic excitation light source (PILL Polychrome V, TILL Photonics) and an Andor iXon DV885 EM CCD camera (TILL Photonics). A 340/380 nm ratio image was generated following background subtraction. Fluorescence emission was quantified using Andor iQ Live cell imaging software (Andor Technology). This experiment has been performed at the UMCG Microscopy and Imaging Center (UMIC), which is sponsored by NWO-grants 40-00506-98-9021 (*TissueFAXS*) and 175-010-2009-023 (*Zeiss 2p*), University of Groningen.

### Isometric tension measurements

Bovine tracheae were obtained from a local slaughterhouse and transported to the laboratory in Krebs-Henseleit (KH) buffer (118 mM NaCl, 5.6 mM KCl, 1.18 mM MgSO_4_, 2.5 mM CaCl_2_, 1.28 mM NaH_2_PO_4_, 25 mM NaHCO_3_, 5.5 mM Glucose, pregassed with 5% CO_2_ and 95% O_2,_ pH 7.4). After dissection of the smooth muscle layer and removal of the mucosa and connective tissue, tracheal smooth muscle strips were prepared while incubated in gassed KH-buffer at room temperature. Strips were cut with a length of 1 cm and width of 2 mm. Tissue strips were washed once in sterile DMEM supplemented NaHCO_3_ (7 mM), HEPES (10 mM), sodium pyruvate (1 mM), non-essential amino acids (GIBCO, #11140-050, 1:100), gentamycin (45 mg/mL), Penicillin-Streptomycin (200 units/mL), antimycotic (2.5 μg/mL) and transferred into suspension culture flasks. Strips were maintained in culture in an incubator shaker (37 °C, 55 RPM) for 3 days.

After washing with KH-buffer, strips were mounted for isometric recording (Grass force-displacement transducer FT03) in 20 mL water-jacketed organ baths, containing KH-buffer at 37 °C, continuously gassed with 5% CO_2_ and 95% O_2_, pH 7.4. During a 90 min equilibration period with washouts every 30 min, resting tension was gradually adjusted to 3 g. Muscle strips were then pre-contracted with 20 and 40 mM isotonic KCl. After washing, tension was re-adjusted to 3 g and cumulative dose response curves were constructed by stepwise increasing the concentration of isotonic histamine.

### Statistical analysis

All data represent the mean ± SEM, of at least three independent experiments. Comparisons between two groups were made using an unpaired Student’s t-test. Comparisons between three or more groups with one independent variable were performed using a one-way ANOVA followed by Dunnett’s or Tukey’s post hoc test. Groups with two independent variables were compared with a two-way ANOVA. A value of p < 0.05 was considered significant, where *represents p < 0.05, **p < 0.01 and ***p < 0.001. Analyses were performed with GraphPad Prism (GraphPad Software, Inc.).

## Additional Information

**How to cite this article**: Koopmans, T. *et al.* Regulation of actin dynamics by WNT-5A: implications for human airway smooth muscle contraction. *Sci. Rep.* **6**, 30676; doi: 10.1038/srep30676 (2016).

## Supplementary Material

Supplementary Information

## Figures and Tables

**Table 1:** Primer sequences used.

Amplicon	Forward sequence (5′→3′)	Reverse sequence (5′→3′)
IP3R	GTGCTTCATCGTCCTGGTGA	TCTCATCCTGGGGAACCAGT
SERCA2	TCGAACCCTTGCCACTCATC	CCAGTATTGCAGGTTCCAGGT
RYR1	CAAGCCCCTTAGTCCCCAAG	GGGGGCGTAGCACAGATTTA
RYR2	AGCTCGTATTAACCGTTGGCT	TTTCATCCCCGATCCCTCCT
RYR3	CCCGATATGAAGTGCGACGA	ATCACCAATGCCACCTCCTG
18S ribosomal RNA	CGCCGCTAGAGGTGAAATTC	TTGGCAAATGCTTTCGCTC
B2M	AAGCAGCATCATGGAGGTTTG	AAGCAAGCAAGCAGAATTTGGA
RPL13A	ACCGCCCTACGACAAGAAAA	GCTGTCACTGCCTGGTACTT

## References

[b1] WHO. Asthma. Available at: http://www.who.int/respiratory/asthma/en/ (2013).

[b2] Bai T. R. Abnormalities in airway smooth muscle in fatal asthma. A comparison between trachea and bronchus. Am. Rev. Respir. Dis. 143, 441–443 (1991). 1990965 10.1164/ajrccm/143.2.441

[b3] Cerrina J. *et al.* Human isolated bronchial muscle preparations from asthmatic patients: effects of indomethacin and contractile agonists. Prostaglandins 37, 457–469 (1989). 2762556 10.1016/0090-6980(89)90095-6

[b4] Chin L. Y. M. *et al.* Mechanical properties of asthmatic airway smooth muscle. Eur. Respir. J. 40, 45–54 (2012). 22267756 10.1183/09031936.00065411

[b5] Ma X. *et al.* Changes in biophysical and biochemical properties of single bronchial smooth muscle cells from asthmatic subjects. Am. J. Physiol. Lung Cell. Mol. Physiol. 283, L1181–1189 (2002). 12388349 10.1152/ajplung.00389.2001

[b6] Matsumoto H. *et al.* Comparison of gel contraction mediated by airway smooth muscle cells from patients with and without asthma. Thorax 62, 848–854 (2007). 17412779 10.1136/thx.2006.070474 PMC2094259

[b7] Sutcliffe A. *et al.* Increased nicotinamide adenine dinucleotide phosphate oxidase 4 expression mediates intrinsic airway smooth muscle hypercontractility in asthma. Am. J. Respir. Crit. Care Med. 185, 267–274 (2012). 22108207 10.1164/rccm.201107-1281OC PMC3402550

[b8] Ijpma G. *et al.* Human trachealis and main bronchi smooth muscle are normoresponsive in asthma. Am. J. Respir. Crit. Care Med. 191, 884–893 (2015). 25695616 10.1164/rccm.201407-1296OC PMC4435453

[b9] Fish J. E., Peterman V. I. & Cugell D. W. Effect of deep inspiration on airway conductance in subjects with allergic rhinitis and allergic asthma. J. Allergy Clin. Immunol. 60, 41–46 (1977). 874208 10.1016/0091-6749(77)90081-1

[b10] Fish J. E., Ankin M. G., Kelly J. F. & Peterman V. I. Regulation of bronchomotor tone by lung inflation in asthmatic and nonasthmatic subjects. J. Appl. Physiol. 50, 1079–1086 (1981). 7228758 10.1152/jappl.1981.50.5.1079

[b11] Lim T. K., Pride N. B. & Ingram R. H. Effects of volume history during spontaneous and acutely induced air-flow obstruction in asthma. Am. Rev. Respir. Dis. 135, 591–596 (1987). 3826885 10.1164/arrd.1987.135.3.591

[b12] Lim T. K., Ang S. M., Rossing T. H., Ingenito E. P. & Ingram R. H. The effects of deep inhalation on maximal expiratory flow during intensive treatment of spontaneous asthmatic episodes. Am. Rev. Respir. Dis. 140, 340–343 (1989). 2764370 10.1164/ajrccm/140.2.340

[b13] Skloot G., Permutt S. & Togias A. Airway hyperresponsiveness in asthma: a problem of limited smooth muscle relaxation with inspiration. J. Clin. Invest. 96, 2393–2403 (1995). 7593627 10.1172/JCI118296 PMC185891

[b14] Benayoun L., Druilhe A., Dombret M.-C., Aubier M. & Pretolani M. Airway structural alterations selectively associated with severe asthma. Am. J. Respir. Crit. Care Med. 167, 1360–1368 (2003). 12531777 10.1164/rccm.200209-1030OC

[b15] Léguillette R. *et al.* Myosin, transgelin, and myosin light chain kinase: expression and function in asthma. Am. J. Respir. Crit. Care Med. 179, 194–204 (2009). 19011151 10.1164/rccm.200609-1367OC PMC2633053

[b16] Woodruff P. *et al.* Hyperplasia of smooth muscle in mild to moderate asthma without changes in cell size or gene expression. Am. J. Respir. Crit. Care Med. 169, 1001–1006 (2004). 14726423 10.1164/rccm.200311-1529OC

[b17] Kuo K. H., Wang L., Paré P. D., Ford L. E. & Seow C. Y. Myosin thick filament lability induced by mechanical strain in airway smooth muscle. J. Appl. Physiol. Bethesda Md 1985 90, 1811–1816 (2001). 10.1152/jappl.2001.90.5.1811 11299271

[b18] Solway J. *et al.* Actin dynamics: a potential integrator of smooth muscle (Dys-)function and contractile apparatus gene expression in asthma. Parker B. Francis lecture. Chest 123, 392S–8S (2003). 12629000 10.1378/chest.123.3_suppl.392s

[b19] Lavoie T. L. *et al.* Disrupting actin-myosin-actin connectivity in airway smooth muscle as a treatment for asthma? Proc. Am. Thorac. Soc. 6, 295–300 (2009). 19387033 10.1513/pats.200808-078RM PMC2677405

[b20] Smolich B., McMahon J., McMahon A. & Papkoff J. Wnt family proteins are secreted and associated with the cell surface. Mol. Biol. Cell 4, 1267–1275 (1993). 8167409 10.1091/mbc.4.12.1267 PMC275763

[b21] Willert K. *et al.* Wnt proteins are lipid-modified and can act as stem cell growth factors. Nature 423, 448–452 (2003). 12717451 10.1038/nature01611

[b22] Nusse R. Wnt signaling in disease and in development. Cell Res. 15, 28–32 (2005). 15686623 10.1038/sj.cr.7290260

[b23] Kumawat K. *et al.* Noncanonical WNT-5A signaling regulates TGF-β-induced extracellular matrix production by airway smooth muscle cells. FASEB J. Off. Publ. Fed. Am. Soc. Exp. Biol. 27, 1631–1643 (2013). 10.1096/fj.12-217539 23254341

[b24] Choy D. *et al.* Gene expression patterns of Th2 inflammation and intercellular communication in asthmatic airways. J. Immunol. Baltim. Md 1950 186, 1861–1869 (2011). 10.4049/jimmunol.1002568 PMC3981556 21187436

[b25] Ault K., Durmowicz G., Galione A., Harger P. & Busa W. Modulation of Xenopus embryo mesoderm-specific gene expression and dorsoanterior patterning by receptors that activate the phosphatidylinositol cycle signal transduction pathway. Dev. Camb. Engl. 122, 2033–2041 (1996). 10.1242/dev.122.7.2033 8681784

[b26] Slusarski D., Corces V. & Moon R. Interaction of Wnt and a Frizzled homologue triggers G-protein-linked phosphatidylinositol signalling. Nature 390, 410–413 (1997). 9389482 10.1038/37138

[b27] Sheldahl L., Park M., Malbon C. & Moon R. Protein kinase C is differentially stimulated by Wnt and Frizzled homologs in a G-protein-dependent manner. Curr. Biol. CB 9, 695–698 (1999). 10395542 10.1016/s0960-9822(99)80310-8

[b28] Kühl M., Sheldahl L., Malbon C. & Moon R. Ca(2+)/calmodulin-dependent protein kinase II is stimulated by Wnt and Frizzled homologs and promotes ventral cell fates in Xenopus. J. Biol. Chem. 275, 12701–12711 (2000). 10777564 10.1074/jbc.275.17.12701

[b29] Kikuchi A., Yamamoto H., Sato A. & Matsumoto S. Wnt5a: its signalling, functions and implication in diseases. Acta Physiol. Oxf. Engl. 204, 17–33 (2012). 10.1111/j.1748-1716.2011.02294.x 21518267

[b30] Gosens R. *et al.* Caveolin-1 is required for contractile phenotype expression by airway smooth muscle cells. J. Cell. Mol. Med. 15, 2430–2442 (2011). 21199324 10.1111/j.1582-4934.2010.01246.x PMC3822954

[b31] Smith P. G., Roy C., Zhang Y. N. & Chauduri S. Mechanical stress increases RhoA activation in airway smooth muscle cells. Am. J. Respir. Cell Mol. Biol. 28, 436–442 (2003). 12654632 10.1165/rcmb.4754

[b32] Sit S.-T. & Manser E. Rho GTPases and their role in organizing the actin cytoskeleton. J. Cell Sci. 124, 679–683 (2011). 21321325 10.1242/jcs.064964

[b33] Kumawat K. *et al.* TGF-β-activated kinase 1 (TAK1) signaling regulates TGF-β-induced WNT-5A expression in airway smooth muscle cells via Sp1 and β-catenin. Plos One 9, e94801 (2014). 24728340 10.1371/journal.pone.0094801 PMC3984265

[b34] Mack C. P., Somlyo A. V., Hautmann M., Somlyo A. P. & Owens G. K. Smooth muscle differentiation marker gene expression is regulated by RhoA-mediated actin polymerization. J. Biol. Chem. 276, 341–347 (2001). 11035001 10.1074/jbc.M005505200

[b35] Cervantes S., Yamaguchi T. P. & Hebrok M. Wnt5a is essential for intestinal elongation in mice. Dev. Biol. 326, 285–294 (2009). 19100728 10.1016/j.ydbio.2008.11.020 PMC2654720

[b36] Mericskay M., Kitajewski J. & Sassoon D. Wnt5a is required for proper epithelial-mesenchymal interactions in the uterus. Dev. Camb. Engl. 131, 2061–2072 (2004). 10.1242/dev.01090 15073149

[b37] Cohen E. D., Miller M. F., Wang Z., Moon R. T. & Morrisey E. E. Wnt5a and Wnt11 are essential for second heart field progenitor development. Dev. Camb. Engl. 139, 1931–1940 (2012). 10.1242/dev.069377 PMC3347685 22569553

[b38] Verkaar F. & Zaman G. J. R. A model for signaling specificity of Wnt/Frizzled combinations through co-receptor recruitment. FEBS Lett. 584, 3850–3854 (2010). 20800062 10.1016/j.febslet.2010.08.030

[b39] Baarsma H. A. *et al.* Activation of WNT/β-catenin signaling in pulmonary fibroblasts by TGF-β_1_ is increased in chronic obstructive pulmonary disease. PloS One 6, e25450 (2011). 21980461 10.1371/journal.pone.0025450 PMC3184127

[b40] Kumawat K. *et al.* Noncanonical WNT-5A signaling regulates TGF-β-induced extracellular matrix production by airway smooth muscle cells. FASEB J. Off. Publ. Fed. Am. Soc. Exp. Biol. 27, 1631–1643 (2013). 10.1096/fj.12-217539 23254341

[b41] Shimizu R. T., Blank R. S., Jervis R., Lawrenz-Smith S. C. & Owens G. K. The smooth muscle alpha-actin gene promoter is differentially regulated in smooth muscle versus non-smooth muscle cells. J. Biol. Chem. 270, 7631–7643 (1995). 7706311 10.1074/jbc.270.13.7631

[b42] Madsen C. S., Hershey J. C., Hautmann M. B., White S. L. & Owens G. K. Expression of the smooth muscle myosin heavy chain gene is regulated by a negative-acting GC-rich element located between two positive-acting serum response factor-binding elements. J. Biol. Chem. 272, 6332–6340 (1997). 9045653 10.1074/jbc.272.10.6332

[b43] Li L., Liu Z., Mercer B., Overbeek P. & Olson E. N. Evidence for serum response factor-mediated regulatory networks governing SM22alpha transcription in smooth, skeletal, and cardiac muscle cells. Dev. Biol. 187, 311–321 (1997). 9242426 10.1006/dbio.1997.8621

[b44] Sotiropoulos A., Gineitis D., Copeland J. & Treisman R. Signal-regulated activation of serum response factor is mediated by changes in actin dynamics. Cell 98, 159–169 (1999). 10428028 10.1016/s0092-8674(00)81011-9

[b45] Mack C. P. Signaling mechanisms that regulate smooth muscle cell differentiation. Arterioscler. Thromb. Vasc. Biol. 31, 1495–1505 (2011). 21677292 10.1161/ATVBAHA.110.221135 PMC3141215

[b46] Morita T., Mayanagi T. & Sobue K. Dual roles of myocardin-related transcription factors in epithelial mesenchymal transition via slug induction and actin remodeling. J. Cell Biol. 179, 1027–1042 (2007). 18056415 10.1083/jcb.200708174 PMC2099179

[b47] O’Connor J. W. & Gomez E. W. Cell adhesion and shape regulate TGF-beta1-induced epithelial-myofibroblast transition via MRTF-A signaling. PloS One 8, e83188 (2013). 24340092 10.1371/journal.pone.0083188 PMC3858353

[b48] Hautmann M. B., Adam P. J. & Owens G. K. Similarities and differences in smooth muscle alpha-actin induction by TGF-beta in smooth muscle versus non-smooth muscle cells. Arterioscler. Thromb. Vasc. Biol. 19, 2049–2058 (1999). 10479645 10.1161/01.atv.19.9.2049

[b49] Hu B., Wu Z. & Phan S. H. Smad3 mediates transforming growth factor-beta-induced alpha-smooth muscle actin expression. Am. J. Respir. Cell Mol. Biol. 29, 397–404 (2003). 12702545 10.1165/rcmb.2003-0063OC

[b50] Valcourt U., Kowanetz M., Niimi H., Heldin C.-H. & Moustakas A. TGF-beta and the Smad signaling pathway support transcriptomic reprogramming during epithelial-mesenchymal cell transition. Mol. Biol. Cell 16, 1987–2002 (2005). 15689496 10.1091/mbc.E04-08-0658 PMC1073677

[b51] Sebe A. *et al.* Transforming growth factor-beta-induced alpha-smooth muscle cell actin expression in renal proximal tubular cells is regulated by p38beta mitogen-activated protein kinase, extracellular signal-regulated protein kinase1,2 and the Smad signalling during epithelial-myofibroblast transdifferentiation. Nephrol. Dial. Transplant. Off. Publ. Eur. Dial. Transpl. Assoc. - Eur. Ren. Assoc. 23, 1537–1545 (2008). 10.1093/ndt/gfm789 18192325

[b52] Gosens R. *et al.* Role of caveolin-1 in p42/p44 MAP kinase activation and proliferation of human airway smooth muscle. Am. J. Physiol. Lung Cell. Mol. Physiol. 291, L523–534 (2006). 16617096 10.1152/ajplung.00013.2006

[b53] Ruijter J. M. *et al.* Amplification efficiency: linking baseline and bias in the analysis of quantitative PCR data. Nucleic Acids Res. 37, e45 (2009). 19237396 10.1093/nar/gkp045 PMC2665230

[b54] Ruijter J. M. *et al.* Evaluation of qPCR curve analysis methods for reliable biomarker discovery: bias, resolution, precision, and implications. Methods San Diego Calif 59, 32–46 (2013). 10.1016/j.ymeth.2012.08.011 22975077

