# Supplementary material for: Regulation of actin dynamics by WNT-5A: implications for human airway smooth muscle contraction
Source: Sci Rep. 2016 Jul 29;6:30676. doi: 10.1038/srep30676 (PMC4965744; doi:10.1038/srep30676)

Regulation of actin dynamics by WNT-5A: implications for human airway smooth muscle contraction

*Tim Koopmans[1,2], Kuldeep Kumawat[1,2], Andrew J Halayko[3], Reinoud Gosens[1,2]

[1]Department of Molecular Pharmacology, University of Groningen, The Netherlands

[2]Groningen Research Institute for Asthma and COPD (GRIAC), University of Groningen, The Netherlands

[3]Department of Physiology and Pathophysiology, University of Manitoba, Canada

Figure 1B

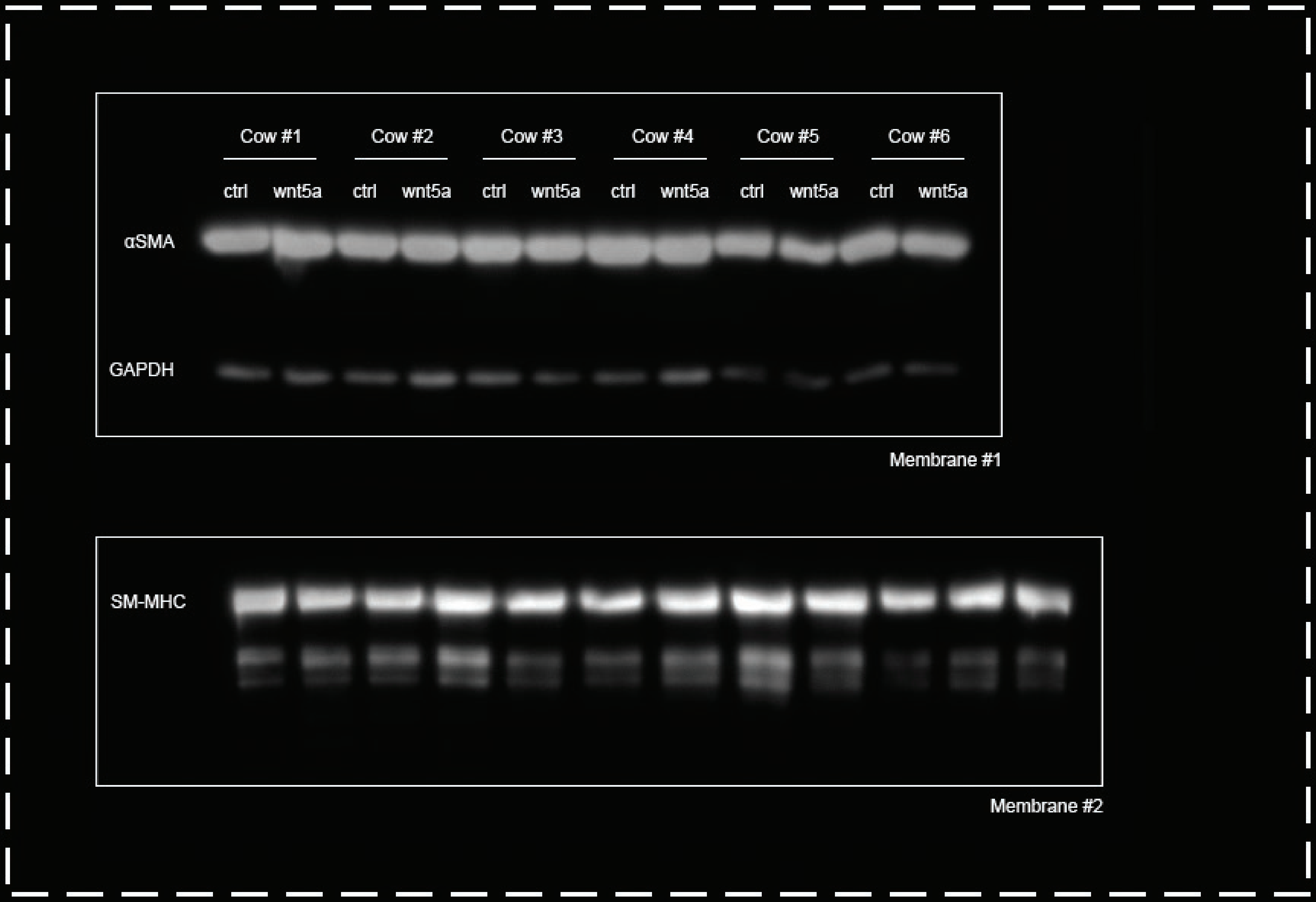
Cow #1
Cow #2
Cow #3
Cow #4
Cow #5
Cow #6
ctrl
wnt5a
αSMA
GAPDH
Membrane #1
SM-MHC
Membrane #2

Figure 3A

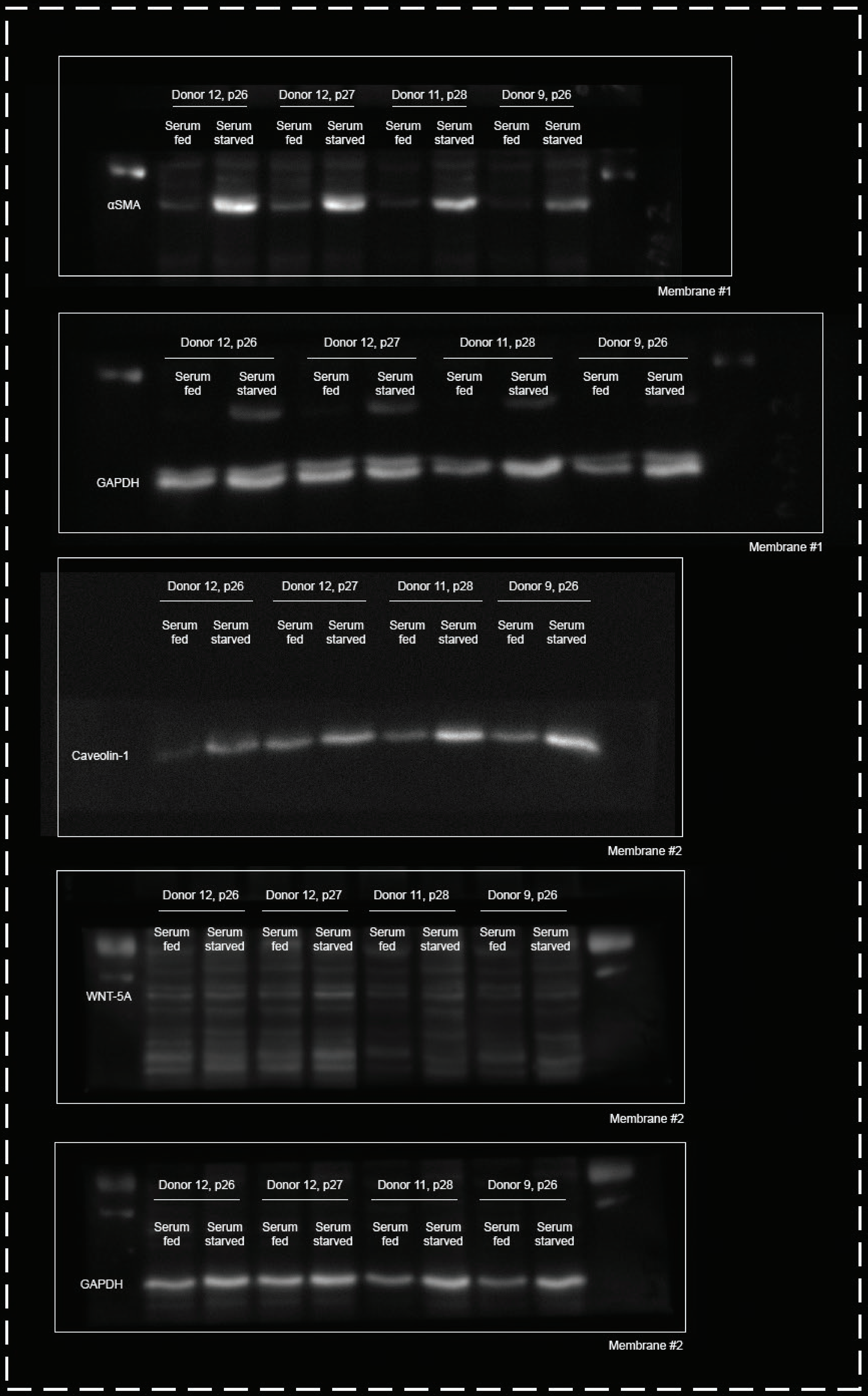

Figure 3B

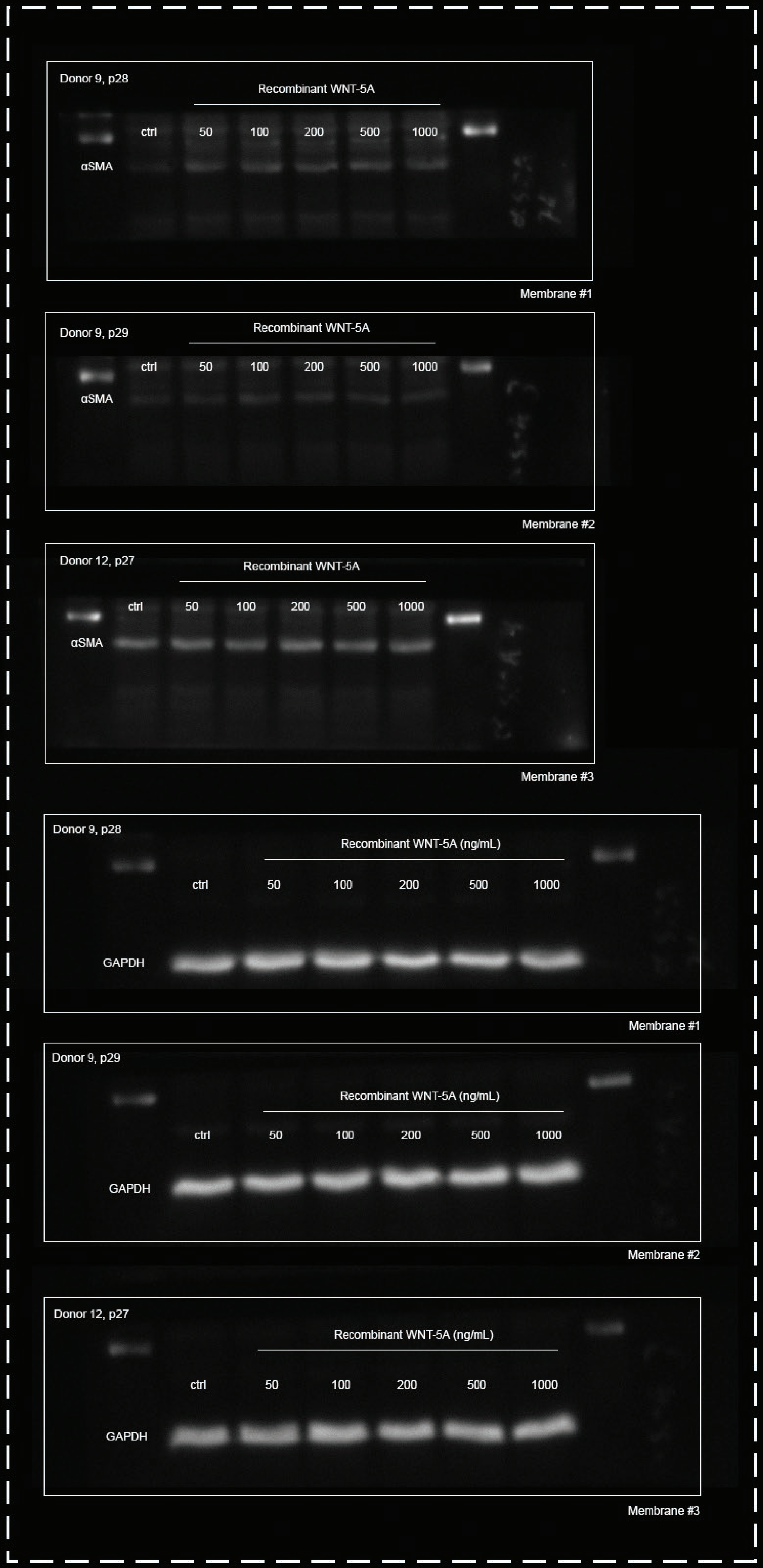

Figure 6A/C

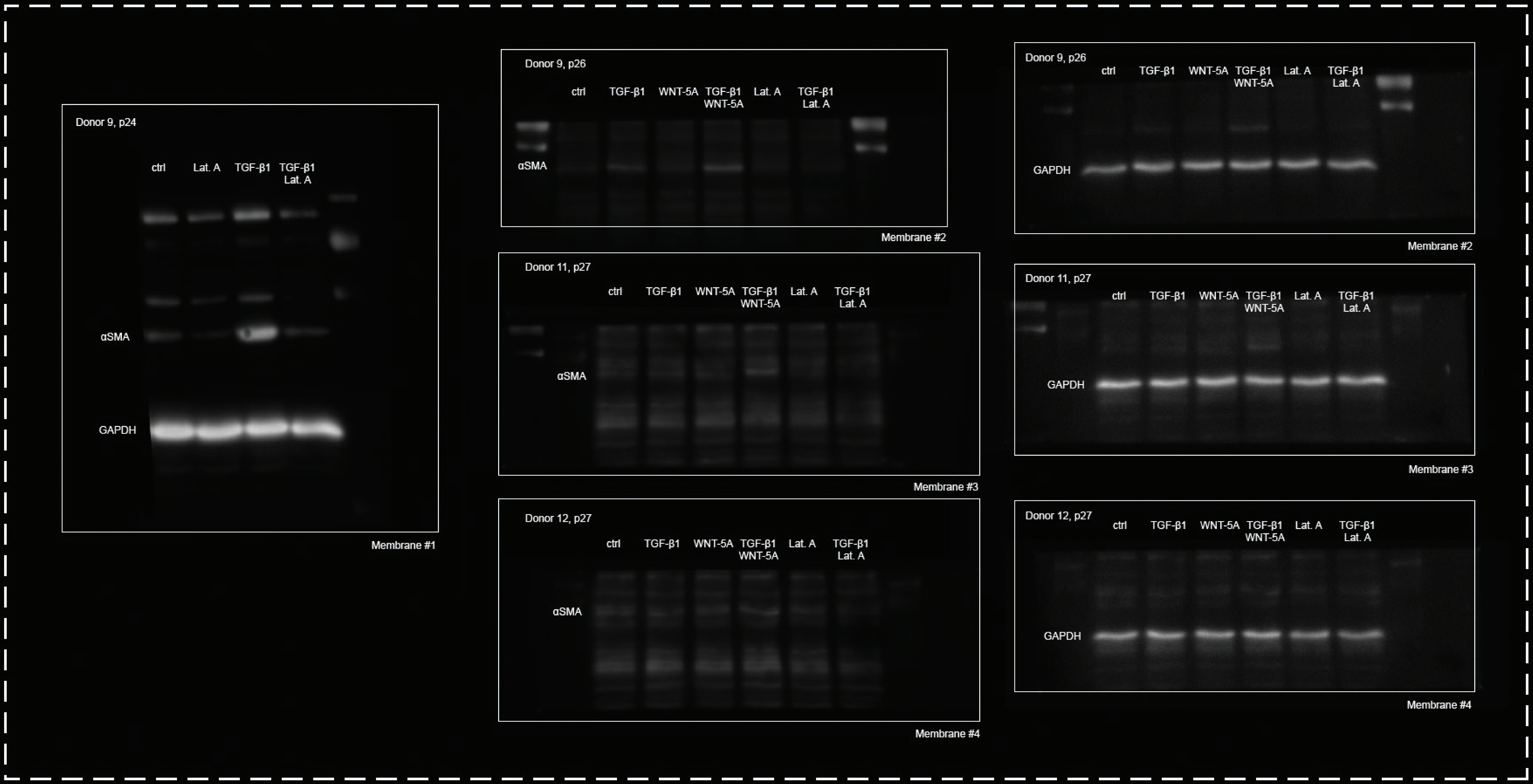

Figure 6B

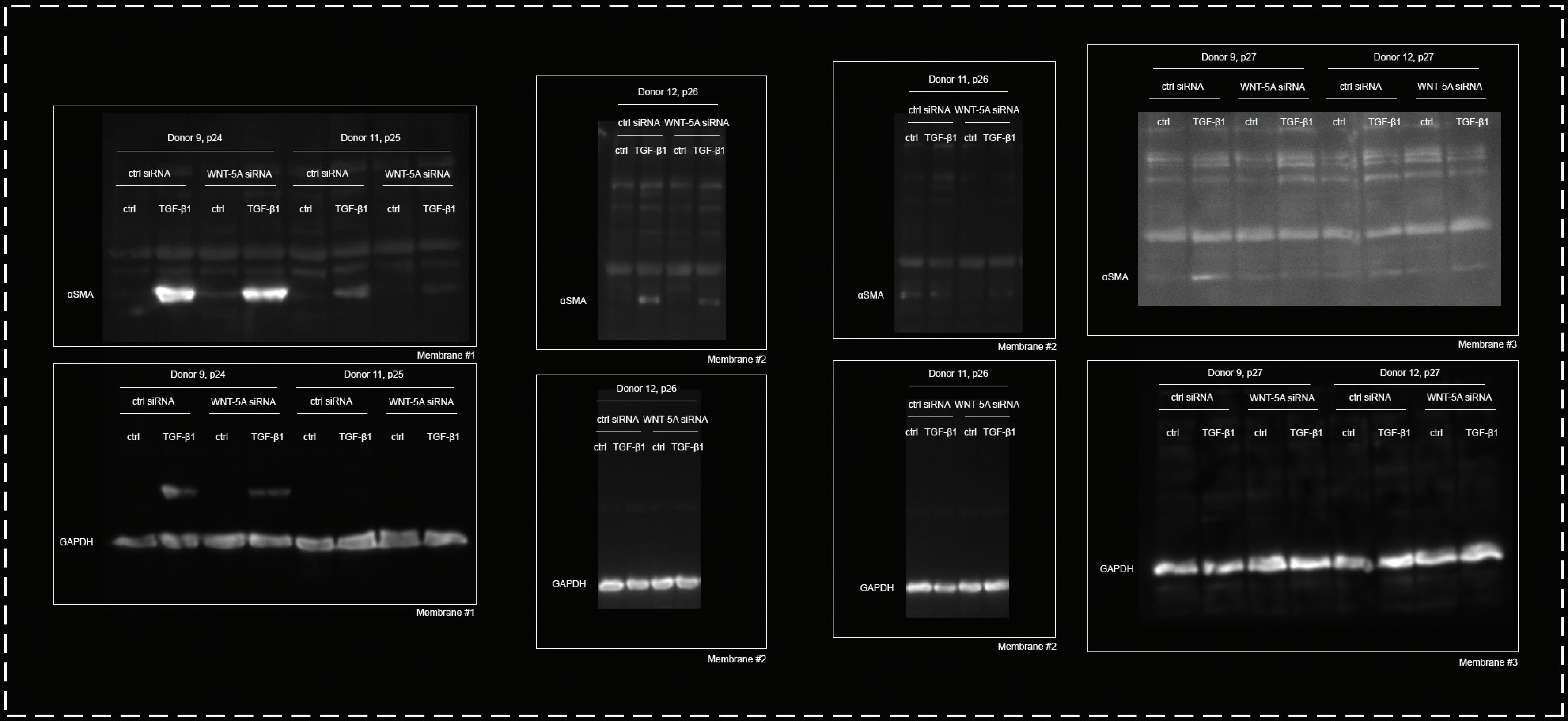

Supplement: Supplementary Information [file srep30676-s1.pdf]
